# Targeting PD-L1 in solid cancer with myeloid cells expressing a CAR-like immune receptor

**DOI:** 10.3389/fimmu.2024.1380065

**Published:** 2024-04-25

**Authors:** Kayla Myers Chen, Daniel Grun, Brian Gautier, Shivaprasad Venkatesha, Michael Maddox, Ai-Hong Zhang, Peter Andersen

**Affiliations:** ^1^ Vita Therapeutics, Baltimore, MD, United States; ^2^ Department of Medicine, Johns Hopkins University School of Medicine, Baltimore, MD, United States

**Keywords:** macrophages, chimeric antigen receptor (CAR), solid cancer, PD-L1, PD-1, phagocytosis, adoptive cell therapy

## Abstract

**Introduction:**

Solid cancers Myeloid cells are prevalent in solid cancers, but they frequently exhibit an anti-inflammatory pro-tumor phenotype that contribute to the immunosuppressive tumor microenvironment (TME), which hinders the effectiveness of cancer immunotherapies. Myeloid cells’ natural ability of tumor trafficking makes engineered myeloid cell therapy an intriguing approach to tackle the challenges posed by solid cancers, including tumor infiltration, tumor cell heterogenicity and the immunosuppressive TME. One such engineering approach is to target the checkpoint molecule PD-L1, which is often upregulated by solid cancers to evade immune responses.

**Method:**

Here we devised an adoptive cell therapy strategy based on myeloid cells expressing a Chimeric Antigen Receptor (CAR)-like immune receptor (CARIR). The extracellular domain of CARIR is derived from the natural inhibitory receptor PD-1, while the intracellular domain(s) are derived from CD40 and/or CD3ζ. To assess the efficacy of CARIR-engineered myeloid cells, we conducted proof-of-principle experiments using co-culture and flow cytometry-based phagocytosis assays in vitro. Additionally, we employed a fully immune-competent syngeneic tumor mouse model to evaluate the strategy’s effectiveness in vivo.

**Result:**

Co-culturing CARIR-expressing human monocytic THP-1 cells with PD-L1 expressing target cells lead to upregulation of the costimulatory molecule CD86 along with expression of proinflammatory cytokines TNF-1α and IL-1β. Moreover, CARIR expression significantly enhanced phagocytosis of multiple PD-L1 expressing cancer cell lines in vitro. Similar outcomes were observed with CARIR-expressing human primary macrophages. In experiments conducted in syngeneic BALB/c mice bearing 4T1 mammary tumors, infusing murine myeloid cells that express a murine version of CARIR significantly slowed tumor growth and prolonged survival.

**Conclusion:**

Taken together, these results demonstrate that adoptive transfer of PD-1 CARIR-engineered myeloid cells represents a promising strategy for treating PD-L1 positive solid cancers.

## Graphical Abstract

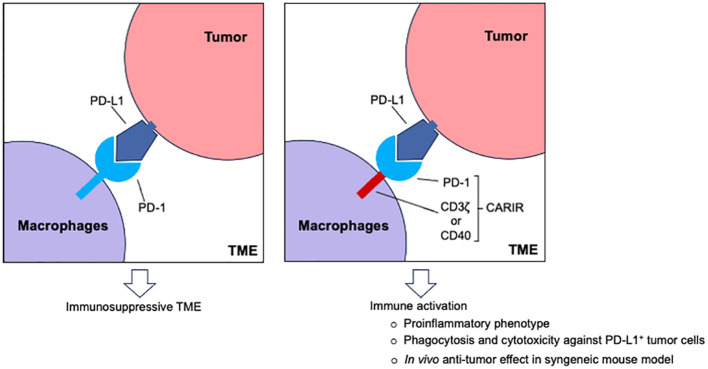

## Introduction

Cancer is a leading cause of death in the US, with solid cancer accounting for nearly 90% of all cases (1). Adoptive cell therapy using chimeric antigen receptor (CAR) T-cells has been tremendously effective against hematological malignancies such as leukemia and lymphomas, but responses in solid tumors have been elusive due to barriers for T cells infiltration, immunosuppressive tumor microenvironment (TME), and inherent tumor cell heterogeneity. Likewise, monoclonal antibody-based PD-1/PD-L1 checkpoint immunotherapies have shown durable responses only in a subset of patients (2), indicating checkpoint inhibition alone is insufficient. Consequently, there is a high demand in developing novel and more effective therapies for solid cancer treatment.

Adoptive transfer of CAR-expressing myeloid cells, including macrophages, has emerged as a promising strategy for treating solid cancer (3, 4). In stark contrast to the limited lymphocyte infiltration, myeloid precursors are actively recruited through cancer-driven myelopoiesis. As a result, macrophages usually are the most abundant immune cells in solid cancer and can account for up to 50% of the tumor volume (5). Being the most functionally versatile innate immune cells in the body, M1 type macrophages can destroy tumor cells themselves and orchestrating an inflammatory adaptive antitumor immune response. However, under the influence of tumor- and non-tumor derived factors in the TME, tumor associated macrophages (TAMs) are often polarized towards an anti-inflammatory, M2-like state that promote tumor growth and metastasis (6). Notably, while the programmed cell death protein 1 (PD-1) is best known as the immune checkpoint receptor expressed on activated T cells, PD-1 expression is associated with TAMs as well (7), indicating that PD-1/PD-L1 checkpoint therapy may exert its effect, in part, via TAMs (7, 8). Since the programmed cell death ligand 1 (PD-L1) is frequently overexpressed in solid cancer as a common pathway of immune evasion (9), it makes PD-L1 a potential target candidate for adoptive myeloid cell therapy.

By designing a PD-L1 specific CAR-like immune receptor (CARIR) for myeloid cells, we demonstrate how myeloid cells could be redirected to targeting PD-L1^+^ solid cancer. Antitumor potency of CARIR modified human macrophages was evaluated by phagocytic activity against PD-L1^+^ tumor cells *in vitro* and using a syngeneic tumor mouse model *in vivo*. Our results demonstrate that CARIR-expressing macrophages have increased phagocytotic activity against PD-L1^+^ cancer cells *in vitro* and that systemic delivery of CARIR-expressing myeloid cells inhibit tumor growth *in vivo*.

## Materials and methods

### Cell lines and mice

THP-1 cell line (Cat# TIB-202) was purchased from ATCC (American Type Culture Collection). Following human or mouse tumor cell lines were purchased from ATCC: NCI-H358 (Cat# CRL-5807), Hs578T (Cat# HTB-126), SK-MEL-28 (Cat# HTB-72), RM-1 (CRL-3310), 4T1(CRL-2539). In addition, MDA-MB-231 cell line was obtained from GeneCopoeia (Cat# SL018). THP-1 cells were cultured in RPMI-1640 medium containing 10% (vol/vol) FBS, 100 µ/ml penicillin, 100 mg/ml streptomycin, and 50μM 2-mercaptoethanol (Thermo Scientific, Cat# 21985023). The tumor cells were cultured in complete medium (RPMI 1640 or DMEM) containing 10% (vol/vol) FBS, 100 U/ml penicillin, 100 ug/ml streptomycin.

Female Balb/c mice (stock no. 006584) between 6-7 weeks old were purchased from The Jackson Laboratory to serve as the syngeneic recipients for 4T1 tumor cell implantation and adoptive myeloid cell therapy. The mice, 5 per cage, were housed in the laboratory of Washington Biotechnology Inc. (Baltimore, MD) in autoclaved solid floor polycarbonate cages with filter-top, supplied with autoclaved bedding, at 22°C with a 12 hours’ light/dark cycle. Balb/c mice of the same sex and age were used to serve as the donor for bone marrow hematopoietic stem cells (HSCs) to generate immature myeloid cells for adoptive cell therapy. All animal experiments were approved by the Institutional Animal Care and Use Committee (IACUC) and conducted in accordance with the Washington Biotechnology, Inc. guidelines.

### Lentiviral vectors and lentiviral transduction

VSV-G pseudotyped third-generation lentiviral vectors encoding for CARIRs were custom ordered from VectorBuilder. Lentiviral vectors encoding human (Cat# LTV0746) or mouse PD-L1 (Cat# LTV1858) containing stable selection marker were purchased from G&P Biosciences. Lentiviral transduction of CARIR or PD-L1 were done in 24-well plate at MOI of 3, or as indicated, in the presence of 1× LentiBOOST (SIRION Biotech SB-P-LV-101-11). Transduction was facilitated through spinoculation by centrifuging the plate at 2500 RPM for 2h at 32°C. For overexpressing PD-L1, 24 hours following transduction, the cells were cultured in the presence of 2 µg/mL of puromycin for 10 days before analyzing PD-L1 expression via flowcytometry.

### Generation of primary myeloid cells or macrophages from human and mouse tissues

For generating human primary macrophages, human mobilized peripheral blood derived CD34^+^ cells (Lonza, 4Y-101C) were expanded in StemSpan SFEM II (StemCell Technologies, 09655), supplemented with 100 ng/mL human recombinant SCF, FLT3-L, TPO (Peprotech, HHCS3), 100 µ/ml penicillin, and 100 mg/ml streptomycin. Macrophages were differentiated from the expanded CD34^+^ cells by first culturing for 7 days in StemSpan SFEM II supplemented with StemSpan Myeloid Expansion Supplement II (StemCell Tech 02694), and then culturing for another 7 days in IMDM media containing 20 ng/mL MCSF (PeproTech, 300-25) and 10% human AB serum (Sigma, H4522).

Mouse myeloid cells were prepared from bone marrow lineage^-^ cells of Balb/C mice. After isolated from the dissected tibia, fibula, and femur bones, the bone marrow cells were stained with biotin mouse lineage panel (BD Biosciences, 559971), followed by anti-biotin microbeads (Miltenyi, 130-105-637), before negative selection of lineage^-^ cells using AutoMACS (Miltenyi). Lineage^-^ cells were expanded in IMDM (Cytiva, SH30228.FS) supplemented with 20% BIT9500 (StemCell Tech, 09500), 100 ng/mL of murine recombinant SCF, FLT3-L, TPO (Peprotech, MHCS3), 100 µ/ml penicillin, and 100 µg/ml streptomycin. Mouse myeloid cells were differentiated in IMDM (Cytiva, SH30228.FS) supplemented with 20% BIT9500 (StemCell Tech, 09500), 20 ng/mL M-CSF (Peprotech, 315-02), 100 U/ml penicillin, and 100 mg/ml streptomycin.

### Flow cytometry

The following monoclonal antibodies (mAbs) and their isotype controls were used for flow cytometry: APC human PD-1, APC human PD-L1, Brilliant Violet human PD-L1, APC human CD86, APC human CD11b, PE human CD247 (CD3z), APC mouse CD11b; PE human EGFR from R&D Systems; APC mouse PD-L1 from Tonbo Biosciences. In addition, following reagents were used in flow cytometry: recombinant human PD-L1 Fc chimera Biotin protein (R&D Systems); APC Streptavidin (Tonbo Biosciences); Zombie NIR viability dye from BioLegend; CellTrace Violet, CellTrace CFSE, and CellTrace Yellow from Invitrogen.

For flow cytometry analysis, cells were resuspended in Cell Staining Buffer (BioLegend) containing Fc receptor blocker (Miltenyi Biotec) and incubated for 10 minutes at 4°C. Then the cells were stained in PBS containing 1:1000 diluted Zombie NIR viability dye (BioLegend) for 30 minutes at room temperature. Finally, the cells were stained with antibodies diluted in Cell Staining Buffer for 20 minutes at 4°C. Cells were washed twice with Cell Staining Buffer between the staining steps and once prior to data acquisition using SONY SA3800 or SONY iD7000 spectral flow cytometer. The flow cytometry data was analyzed using FlowJo software.

### ELISA

Supernatant were collected at 72h following the co-culture of the engineered THP-1 macrophages and RM-1 target cells. The levels of human TNF-α, IL-1 β, and IL-6 were measured by ELISA using the DuoSet ELISA Kits (DY210-05, DY201-05, and DY206-05) from R&D Systems per the manufacturer’s protocol. The quantification was based on the OD values at 450 nm measured by a microplate reader (Multiskan Skyhigh, Thermo Scientific), subtracted by the readings at 540 nm.

### *In vitro* phagocytosis assay

Prior to initiating the coculture, macrophages were labeled with CellTrace Violet (Invitrogen C34557) and target cells were labeled with 1:1000 diluted CellTrace CFSE (Invitrogen C34554) or CellTrace Yellow (Invitrogen C34567) for 30 minutes at 37 °C. In some conditions, macrophages were pre-treated with 2μM cytochalasin D (Cayman Chemical 11330), 10μg/mL anti-PD1 antibody (BioXCell SIM 0010) or 10μg/mL human IgG4 isotype control antibody (BioXCell CP147) prior to co-culture with target cells. Macrophages and target cells were cocultured at a 5:1 E:T ratio for 3 hours at 37 °C in ultra-low attachment 96-well plates (Corning 7007), unless otherwise indicated. Cells were stained for viability with Zombie NIR (BioLegend 423106) and in some experiments with APC-PD-1 antibody (BioLegend 329908). Data was acquired on SONY SA3800 spectral flow cytometer and analyzed using FlowJo software.

### Flow cytometry-based cytotoxicity assay

To evaluate CARIR-mediated killing activity on PD-L1^+^ target cells, non-modified or CARIR engineered THP-1 cells (2.5 × 10^4^) were co-cultured with either RM-1^hPD-L1^ or RM-1 target cells (5 × 10^3^) in the presence of 5ng/ml PMA in 96-well round-bottom culture plate for 3 days. Following the co-culture, the cells were detached by Accutase (Stemcell Technologies) treatment at 37°C for 5 minutes. The cells were stained with Brillian Violet 605 anti-human PD-L1, APC anti-human CD11b, and Zombie NIR viability dye. Absolute counting beads (BioLegend) were added before data acquiring using SONY iD7000 spectral flow cytometry. The data was analyzed using flowjo software, and the percentage and absolute number of the remaining live tumor cells following the co-culture were compared between the groups.

### Subcutaneous 4T1 Tumor mouse model

Therapeutic effect of the engineered myeloid cells was tested in syngeneic Balb/c mice bearing subcutaneously implanted 4T1 breast cancer. The experiment was conducted in a blinded manner. On day -10, female Balb/c mice were subcutaneously injected on right flank with 5×10^4^ 4T1 T cells in 0.1ml PBS containing 20% Matrigel (Cat# 356231, Corning). On day 0, after the tumor became palpable, 24 of the mice were randomized into 3 groups (n = 8) based on tumor size. Each cage houses one mouse from each of the groups. On day 0, 7, and 14, the mice were treated intravenously through tail vein with 0.2ml PBS containing 1×10^7^ unmodified (WT-M) or CARIR engineered (CARIR-M) mouse bone marrow-derived myeloid cells, or PBS vehicle control. Body weight and tumor growth of the mice were measured 2-3 times a week. A digital caliper (Cat# 500-196-30, MSI Viking) was used for measuring the tumor size, and the tumor volume was calculated using the formula: Tumor volume = ½ (width × width × length). The study endpoints include tumor size beyond 2,000mm^3^, or when tumor growth causes more than 20% body weight loss, or when tumor becomes ulcerated.

### Statistics

Data for *in vitro* studies are representative of a minimum of 2 independent repeats, unless otherwise noted. The *in vitro* data are shown as mean ± SEM, with technical replicates plotted as individual data points. Data for *in vivo* study are shown as mean ± SEM with 8 mice per group. Statistical significance was determined by unpaired *t*-test or one-way ANOVA as indicated using GraphPad Prism software. For all statistical analysis, * indicating *p* < 0.05, ** *p* < 0.01, *** *p* < 0.001, and **** *p* < 0.0001.

## Results

### Stimulation through CARIR causes upregulation of co-stimulatory molecules and production of proinflammatory cytokines in human monocytic THP-1 cells

**Figure 1:**
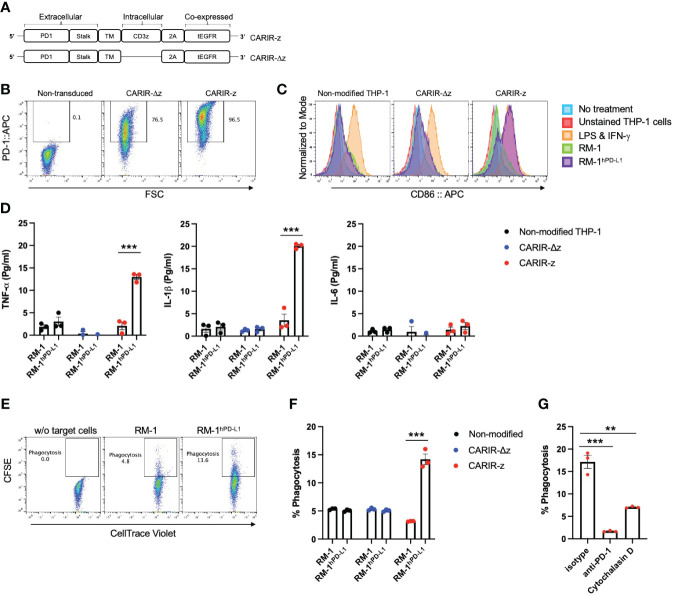
Functional expression of CARIR in human monocytic THP-1 cells. **(A)** Schematic illustration of lentiviral vector-encoded CARIR transgenes, with (CARIR-z) or without an intracellular signaling domain derived from human CD3ζ (CARIR-Δz). PD1 (extracellular portion of programmed cell death protein 1), TM (transmembrane domain), 2A (2A cleavage peptide), tEGFR (truncated extracellular portion of the Epidermal Growth Factor Receptor containing domain III and IV). **(B)** CARIR expression in transduced human monocytic THP-1 cells based on flow staining of human PD-1. THP-1 cells were transduced with lentiviral vector encoding PD-1 CARIR at MOI of 3, and flow analysis was conducted 2 days following the lentiviral transduction. **(C)** The histogram shows the upregulation of co-stimulatory molecule CD86 in CARIR-z transduced THP-1 macrophages following co-culture with RM-1^hPD-L1^ cells. Non-transduced (non-modified) or CARIR transduced THP-1 cells were stimulated with 1ng/ml PMA for 24 hours, followed co-culture for 3 days as indicated. The CD86 expression was analyzed by flow cytometry, and the cells were gated on THP-1 cells based on the characteristic FSC/SSC parameters of the cells. **(D)** Cytokines levels measured by ELISA in the culture supernatant of the experiment as described in **(C)** above. **(E)** Representative flow cytometry dot plots depicting the phagocytosis events appeared in the Q2 quadrant, which is double positive for CellTrace CFSE labeled target cells and CellTrace Violet labeled THP-1 macrophages. Non-modified or CARIR transduced THP-1 cells were treated with 5ng/ml PMA for 24 hours to differentiate the cells toward macrophages, followed by co-culture with RM-1 or RM-1^hPD-L1^ at E:T ratio of 5:1 for 3 hours. Both the effectors and the target cells were fluorescent dye labeled before setting up the co-culture. The cells were gated on FSC/SSC, singlets, and live THP-1 macrophages (CellTrace Violet^+^). **(F)** Bar graph summarizes the % phagocytosis during the 3-hour co-culture, in experiment as described for **(F)**. **(G)** BAR graph shows the inhibition on phagocytosis in the presence of anti-PD-1 blockade antibody or cytochalasin D in experiment as described for **(F)**. The data were expressed as mean ± SEM. **p < 0.01 and ***p < 0.001, by non-paired student t test with 2-tailed distribution. Data shown are representatives of at least 3 independent repeats **(B, C, E)**.

To direct macrophages to target the immune checkpoint molecule PD-L1, which is commonly expressed in many types of solid cancers (9), we designed a CAR-like immune receptor (CARIR), where the extracellular domain is derived from the PD-1 instead of a single chain fragment variable (ScFv) antibody. The intracellular signaling domain is derived from CD3ξ (CARIR-z) like a typical 1^st^ generation CAR design. A CARIR vector without the CD3ξ signaling domain was generated to serve as a control (CARIR-Δz) (**Figure 1A**). Lentiviral vectors were constructed to efficiently deliver the CARIR transgenes into human monocytic THP-1 cells. As shown in **Figure 1B**, 76.5 and 96.5% transduction efficiency were achieved at MOI of 3 for CARIR-Δz and CARIR-z, respectively, determined by staining for PD-1. A truncated version of EGFR (tEGFR) was incorporated in the lentiviral vector to serve as a potential safety module and kill switch, which could be detected in either CARIR-Δz and CARIR-z transduced THP-1 cells (**Supplementary Figure 1A**). In addition, CD3ζ expression is confirmed in the CARIR-z transduced THP-1 cells (**Supplementary Figure 1B**). Next, we confirmed that surface expressed CARIR in CARIR-z engineered THP-1 cells binds human PD-L1 following incubation with biotinylated recombinant human PD-L1 (**Supplementary Figure 2**).

The surface expressed CARIR is functional, as stimulation with plate-bound anti-PD-1 led to an upregulation of co-stimulatory molecules CD86 and CD80 in CARIR-z-THP-1 cells (**Supplementary Figure 3**). To facilitate the functionality test for CARIR modified macrophages, we generated a surrogate PD-L1-expressing model system by stably transducing murine RM-1 cells with human PD-L1 (RM-1^hPD-L1^) (**Supplementary Figure 4**). Next, we differentiated unmodified, CARIR-Δz, and CARIR-z transduced THP-1 cells towards macrophages through PMA stimulation for 24 hours followed by co-culture with WT RM-1 or RM-1^hPD-L1^ cells. Following 3 days of co-culture, THP-1 cells were analyzed for expression of the co-stimulatory molecule CD86 by flow cytometry. As expected, following 3 days of co-culture, CD86 was upregulated in CARIR-z expressing THP-1 cells but not in CARIR-Δz expressing THP-1 cells. Co-culture with unmodified RM-1 cells did not lead to significant change of CD86 expression (**Figure 1C**). In addition, we analyzed the prototypical M1 inflammatory cytokines TNF-α, IL-1β and IL-6 in the supernatant from the co-culture. The production of proinflammatory cytokines, including TNF-α and IL-1β, were significantly increased in CARIR-z expressing THP-1 (**Figure 1D**).

Next, we sought to determine if activation through CARIR-z enhances the macrophage phagocytic activity. To do this, unmodified, CARIR-Δz, and CARIR-z transduced THP-1 derived macrophages were co-cultured with either RM-1 or RM-1^hPD-L1^ for 3 hours before evaluation by flow cytometry for phagocytosis events (**Figure 1E**). As expected, co-culture with RM-1 cells did not increase phagocytosis while co-culture with RM-1^hPD-L1^ led to a 2.8-fold increase (14.2 versus 5.1%) of the % phagocytosis against the target cells by the CARIR-z-THP-1 macrophages. In the contrast, CARIR-Δz expression in THP-1 macrophages did not increase phagocytosis against RM-1^hPD-L1^ target cells (**Figure 1F**). As expected, there was no increase of % phagocytosis by CARIR-z expression in THP-1 macrophages in the presence of the actin polymerization inhibitor, cytochalasin D. Notably, the increase of phagocytosis against PD-L1^+^ target cells by CARIR-z-THP-1 macrophages were CARIR-dependent, since the increase in phagocytosis was completely obligated in the presence of monoclonal anti-PD-1 blockade antibody (**Figure 1G**). Taken together, these results demonstrate that CARIR-mediated activation enhances phagocytosis and polarize macrophages towards a proinflammatory phenotype.

### CARIR expression enables specific killing of PD-L1^+^ target tumor cells by THP-1 macrophages

**Figure 2:**
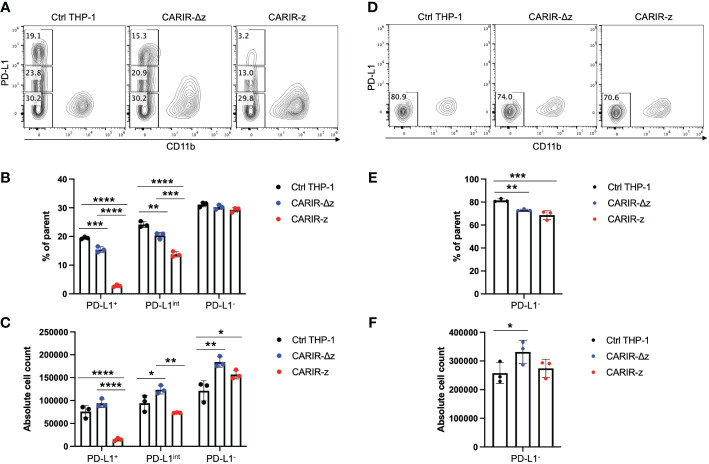
CARIR expression in THP-1 macrophages enabled significant cytotoxicity against PD-L1^+^ target cells. Non-modified- (Ctrl), CARIR-Δz, or CARIR-z-engineered THP-1 effector cells were co-cultured in the presence of 5ng/ml PMA for 3 days with RM-1^hPD-L1^ or WT-RM-1 target cells at effector to target ratio of 5:1. Following the co-culture, the cells were stained with APC anti-human CD11b, Brilliant Violet 605 anti-human PD-L1, and Zombie NIR viability dye. The number of the remaining live CD11b^-^ target cells following the co-culture was quantified by flow cytometry with the use of absolute counting beads. **(A)** Representative contour plots of triplicate experiments show the percentage change for PD-L1^+^, PD-L1^int^, and PD-L1^-^ RM-1^hPD-L1^ tumor cells following the co-culture. **(B)** BAR graphs summarize the percentage data shown on **(A)**. **(C)** BAR graphs summarize the absolute cell count data shown on **(A)**. **(D)** Representative contour plots of triplicate experiments show the percentage change of RM-1 tumor cells (PD-L1^-^) following the co-culture. **(E)** BAR graph summarizes the percentage data shown on **(D)**. **(F)** BAR graph summarizes the absolute cell count data shown on **(D)**. Tumor cells were gated on non-beads, live, singlets, and CD11b^-^. Data were presented as mean ± SEM. *p < 0.05, **p < 0.01, ***p < 0.001, and ****p < 0.0001 by one-way ANOVA.

To determine if CARIR expression could direct macrophages to kill PD-L1^+^ tumor cells, A flow-based cytotoxicity assay was used. THP-1 macrophages were served as the effector cells and RM-1^hPD-L1^ or RM-1 tumor cells as the target cells. Since monocytic THP-1 cells are commonly differentiated into macrophages with PMA treatment, to streamline the assay, we co-cultured the effector cells and the target cells in the presence of PMA. PMA treatment had no significant effect on the viability of either RM-1^hPD-L1^ or RM-1 tumor cells (**Supplementary Figure 5A**). The target RM-1 cells were distinguished from the effector THP-1 cells by their lack of expression of the myeloid marker CD11b (**Supplementary Figure 5B**). Notably, RM-1^hPD-L1^ cells exhibited three distinguished populations based on PD-L1expression levels: CD11b^-^PD-L1^+^, CD11b^-^PD-L1^int^ and CD11b^-^PD-L1^-^ (**Supplementary Figure 5B**). This allowed us to investigate the impact of varying PD-L1 expression levels on target cells in the flow cytometry-based cytotoxicity assay. Compared to control or CARIR-∆z-THP-1, co-culture with CARIR-z-THP-1 led to a significant loss of the PD-L1^+^ and PD-L1^int^, but not the PD-L1^-^ RM-1-^hPD-L1^ cells, with more pronounced elimination observed in the CD11b^-^PD-L1^+^ compared to the CD11b^-^PD-L1^int^ population (**Figures 2A-C**). Conversely, CARIR-Δz-THP-1 notably increased proliferation of PD-L1^int^ and PD-L1^-^ RM-1-^hPD-L1^ cells (**Figures 2A-C**). The absence of cytotoxicity of CARIR-z-THP-1 on PD-L1^-^ target cells was confirmed when non-modified RM-1 cells were used as the target cells (**Figures 2D, E**). These findings suggest that the CD3 zeta signaling domain is essential, and there exists a threshold for PD-L1 expression levels required for robust CARIR-mediated killing by engineered THP-1 macrophages.

### CARIR expression in primary human macrophages increases phagocytosis against PD-L1^+^ target cells

**Figure 3:**
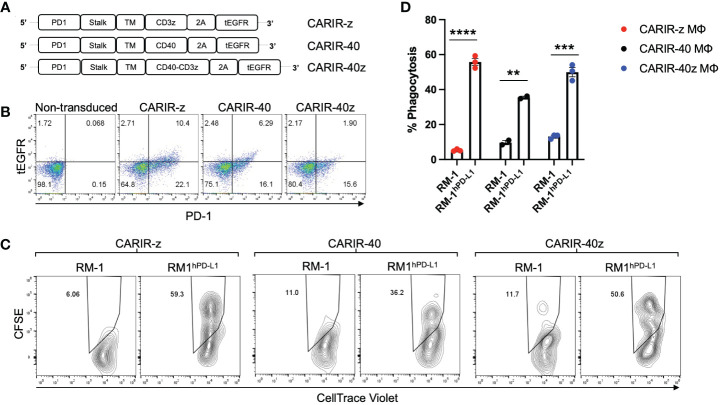
CARIR expression in primary human macrophages enhanced phagocytosis against human PD-L1^+^ target cells. **(A)** Schematic illustrations show different CARIR constructs varying in the intracellular signaling domain(s). **(B)** Flow dot plots show the efficiency of CARIR transduction in human HSCs based on staining for PD-1. **(C)** Representative flow plots showing the % phagocytosis of CARIR expressing primary human macrophage against RM1 or RM1^hPD-L1^ target cells. For generating CARIR modified human macrophages to serve as the effector cells, human CD34^+^ hematopoietic stem cells (HSCs) were engineered to express CARIR through lentiviral transduction, and then differentiated into macrophages. Fluorescent dye labeled effector cells and target cells were co-cultured for 3 hours before flow analysis for the % phagocytosis. The cells were gated on FSC/SSC, singlets, live, and PD-1^+^Violet^+^ macrophages. The CellTrace Violet and CFSE double positive population represent macrophages that have phagocytosed target cells. Data shown are representatives of triplicate experiments **(B, C)**. **(D)** The bar graph summarizes the flow data shown in **(C)** **p < 0.01, ***p < 0.001, and ****p < 0.0001 by unpaired student *t* test with two tailed distributions.

Next, we tested the functionality of CARIR in primary human macrophages, prepared from mobilized peripheral blood derived CD34^+^ hematopoietic stem cells (HSCs). For CARIR, in addition to CARIR-z, we created additional lentiviral constructs with the intracellular signaling domain derived from CD40 (CARIR-40) or from both CD40 and CD3 (CARIR-40z) (**Figure 3A**). HSCs were transduced with lentiviral vector for either CARIR-z, CARIR-40, or CARIR-40z. The transduction efficiencies were between 17 – 32% as determined by PD-1 expression 2 days following the transduction (**Figure 3B**). The transduced cells were expanded and sequentially differentiated towards macrophages. The phagocytosis assay was performed by co-culturing these modified human primary macrophages with RM-1 or RM-1^hPD-L1^ target cells for 3 hours. Consistent to the result obtained with CARIR-z THP-1 macrophages, following 3 hours of co-culturing, CARIR-z modified human primary macrophages had a 10-fold increased phagocytosis against RM-1^hPD-L1^ than RM-1 targets (55.8% vs 5.2%). Similarly, CARIR-40 or CARIR-40z expression in human primary macrophages also led to significant increase in phagocytosis against RM-1^hPD-L1^ than RM-1 targets (35.5 vs 9.7% for CARIR-40; 49.9 vs 13.1% for CARIR-40z) (**Figures 3C, D**). These results further confirmed the functionality of CARIR expression in macrophages in enhancing phagocytosis against human PD-L1^+^ target cells. In addition, the results indicated that CD3ζ signaling domain is sufficient for the CARIR-mediated functionality. Therefore, in the rest of the study we will be focusing on testing CARIR functionality in the format of CARIR-z configuration.

### CARIR expression in human THP-1 macrophages enhances phagocytosis against PD-L1^+^ human tumor cell lines

**Figure 4:**
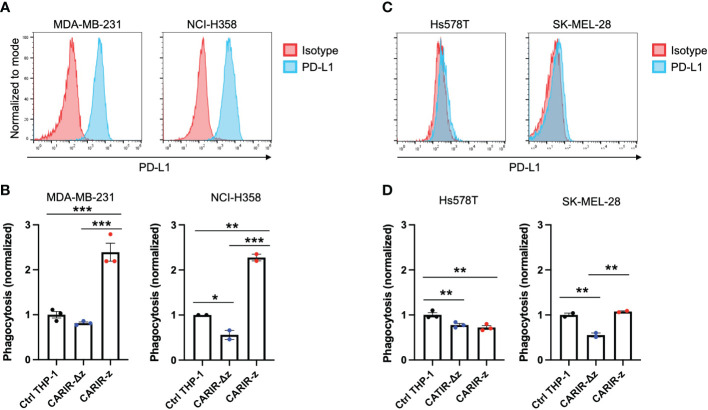
CARIR expression in human THP-1 macrophages increased phagocytosis against PD-L1^+^ human tumor cells. **(A, C)** Histogram shows the detection of cell surface PD-L1 expression by flow cytometry in cultured human tumor cell lines. Data shown are representatives of 3 independent experiments. **(B, D)** Phagocytosis activity against PD-L1^+^ tumor cell lines MDA-MB-231 and NCI-H358 **(B)**, or PD-L1^-^ tumor cell lines Hs578T and SK-MEL-28 **(D)**. Human monocytic THP-1 cells were either nonmodified (Ctrl THP-1) or engineered to express either CARIR-Δz or CARIR-z through lentiviral transduction, and then differentiated toward macrophages by culturing in the presence of 1 ng/ml PMA for 24 hours. CellTrace Violet labeled THP-1 effectors were co-cultured with CellTrace Yellow labeled indicated tumor cells for 4 hours, followed by flow cytometry analysis of the % phagocytosis. Cells were gated on live, singlets, and violet^+^ cells. The events that were double positive for CellTrace Violet and CellTrace Yellow were considered as phagocytic events. The phagocytosis activity was normalized to the non-modified THP-1 condition. The data was presented as mean ± SEM. *p < 0.05, **p < 0.01, and ***p < 0.00 by one-way ANOVA.

Next, instead of artificial human PD-L1 engineered target cells, we asked if PD-L1^+^ human solid tumor cells could be targeted by CARIR modified macrophages *in vitro*. To this goal, we utilized 4 human solid tumor cell lines, including MDA-MB-231, NCI-H358, Hs578T, and SK-MEL-28, to serve as the target cells. The former two lines are PD-L1^+^, while the other two lines were barely detectible for PD-L1 surface expression by flow cytometry (**Figures 4A, C**). *In vitro* phagocytosis assays were performed using each of the above tumor cells as the targets and non-modified or engineered THP-1 macrophages as the effectors. CARIR-z THP-1 had significantly increased phagocytosis activity against both the PD-L1^+^ tumor cell lines, the triple negative breast cancer (TNBC) line MDA-MB-231 and non-small-cell lung cancer (NSCLC) line NCI-H358, as compared to either the nonmodified or CARIR-Δz-THP-1 macrophages conditions. In the contrast, CARIR expression in THP-1 macrophages did not lead to an increased phagocytosis activity against the PD-L1 negative Hs578T and SK-MEL-28 tumor cell lines (**Figures 4B, D**).

### Adoptive transfer of CARIR modified myeloid cells ameliorated tumor growth in a syngeneic TNBC tumor mouse model

**Figure 5:**
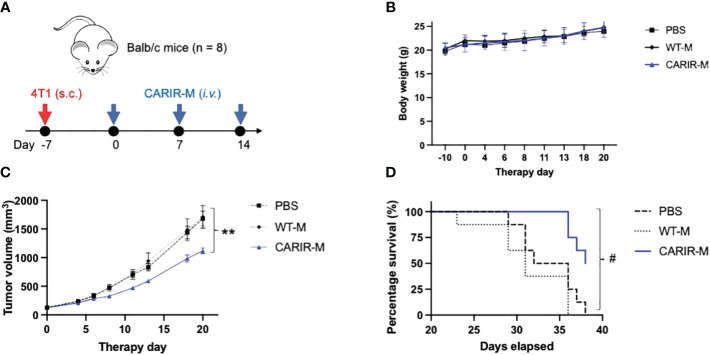
Adoptive transfer of CARIR modified myeloid cells (CARIR-M) slows 4T1 tumor growth and prolongs survival. **(A)** Schematic timeline for the animal experiment. Syngeneic Balb/c mice (8 mice/group) were subcutaneously implanted with 5 × 10^4^ 4T1 breast cancer cells on day -7. Starting on day 0, the mice were injected through the tail vein with 3 weekly doses of 10 × 10^6^ either CARIR-M or control non-modified myeloid cells (WT-M). An additional control group of mice were treated with PBS. Body weight **(B)**, tumor volume **(C)**, and probability of survival **(D)** were measured 2-3 times per week. Data are presented as mean ± SEM. **p < 0.01: CARIR-M vs WT-IMC or PBS by type II ANOVA. #p < 0.05 (CARIR-M vs PBS) and p < 0.01(CARIR-M vs WT-M) by Gehan- Breslow-Wilcoxon test.

Since PD-1 CARIR is specific to PD-L1, CARIR modified macrophage approach can be considered as a cell therapy version of immune checkpoint therapy, enhanced by the chimeric receptor technology. Next, we sought to evaluate the anti-tumor functionality of CARIR *in vivo* using syngeneic Balb/c mice bearing aggressive 4T1 triple negative breast cancer (TNBC). 4T1 tumor expresses positive albeit low cell surface PD-L1 and normally does not respond to classic anti-PD-L1 checkpoint blockade (10). Thus, we prepared a lentiviral vector encoding a murine analogue of human PD-1 CARIR-z. CARIR engineered myeloid cells were prepared by transducing donor Lin^-^ HSCs with the mouse version of CARIR-z. A 48% mouse PD-1 expression (CARIR transduction efficiency) was achieved at the MOI of 10. After a total of 6 days expansion in the presence of SCF, TPO and Flt3L, the transduced cells were further myeloid differentiated in M-CSF medium for 24 hours. In mice with established 4T1 tumor, weekly infusion of CARIR-z myeloid cells (CARIR-M) significantly slowed tumor growth and prolonged survival (**Figure 5**). Of note, no significant differences were observed in body weight measurements between the CARIR-M, WT-M, or the non-treated (PBS) group, suggesting a lack of overt toxicity associated with CARIR-M treatment (**Figure 5B**).

## Discussion

Macrophages are highly plastic in their functionality and have capacity not only in infiltrating solid tumor, but also in tumor cell phagocytosis and orchestrating adaptive immune response through tumor antigen presentation. Adoptive cell therapies using chimeric receptor engineered macrophages or its immediate precursor, monocytes, are becoming an exciting avenue in developing effective treatments for solid cancers (3, 11, 12). However, devising a traditional CAR approach to target tumor-specific antigens remains a significant challenge as unique and broadly expressed tumor-specific antigens are rare. Moreover, solid cancers are heterogenous in nature and targeting a single tumor antigen often leads to antigen escape. As a common mechanism of immune evasion, solid tumor cells and/or tumor-infiltrating immune cells upregulate the immune checkpoint molecule PD-L1 (13). As a result, several studies have described targeting PD-L1 using CAR-T (14–18) or CAR-NK (19) as cell therapy strategies for cancer. To our knowledge, we have demonstrated in this study for the first time that myeloid cells or macrophages that modified to express CARIR could be used to effectively target PD-L1 in solid cancer.

As a chimeric receptor, CARIR is akin to the previously described PD-1-CD28 fusion receptor or PD-L1-specific chimeric switch receptor (CSR) for T cells (20–22). The common feature of these chimeric receptors is that the extracellular domain is derived from the immune checkpoint molecule PD-1. However, the intracellular domain usage and the functional utility of these chimeric receptors differ. The only cytosolic domain of PD-1-CD28 fusion receptor or PD-L1-specific CSR is derived from the costimulatory molecule CD28, and the purpose of expressing these chimeric receptors is to enhance the functionality of tumor-specific TCR-T or CAR-T cells (20–22). In the present study, however, the purpose of the CARIR is to direct macrophages to recognize and attack PD-L1^+^ solid cancer cells and reprogram immunosuppressive TME. Thus, the intracellular region of CARIR is derived from the cytosolic signaling domain(s) of CD3z and/or CD40. As expected, CARIR-z, but not CARIR-Δz, induced a proinflammatory phenotype of THP-1 macrophages and increased phagocytosis and killing activities against RM-1^hPD-L1^ target cells, whereas no effect was seen against WT RM-1 (**Figures 1**, **2**). Although exclusively expressed in T cell lineages, the signaling domain of CD3ζ in a chimeric receptor format is known to be able to transmit signal and trigger phagocytosis in macrophages (11). In this study we also tested a CARIR version with the macrophage specific CD40 signaling domain. However, based on phagocytosis efficiency of PD-L1^+^ target cells (**Figures 3C, D**), CD3ζ outperformed CD40 and CD40/CD3z, which let us to focus our study on the CARIR-z version. It is of note, high expression of PD-L1 has also been found in myeloid derived suppressor cells (MDSC) and subsets of TAMs (7), making them all attractive targets in treating solid cancer (23).

Under physiological condition, PD-1/PD-L1 signaling functions as a mechanism for maintaining immune tolerance, preventing excess immune cell activity that can lead to autoimmunity and tissue damage. This immune-inhibiting axis is exploited by many types of malignancies to evade the body’s anti-tumor immune response. Targeting PD-L1 tilts the immune homeostasis from immune tolerance towards anti-tumor cytotoxicity. As seen with the remarkably successful immune checkpoint therapies, certain level of off-tumor cytotoxicity is inevitable and is expected for CARIR modified macrophages therapy as well. However, there are a few reasons that may argue against a major safety concern for the application of CARIR, which utilizes natural PD-1 domain instead of ScFv antibody for target recognition: 1) the affinity between human PD-1 (CARIR) and its ligand PD-L1 is about 7.2 μM (24), which is several logs lower than clinical approved anti-PD-1/PD-L1 monoclonal antibodies (25), suggesting CARIR modified macrophages might be able to better discriminate between upregulated PD-L1 in the case of solid cancer and the physiological expression of PD-L1 in normal healthy cells; 2) the result from our flow-based killing assay in **Figure 2** indicates a threshold on PD-L1 expression levels on target cells may exist for CARIR-mediated killing activity by the engineered THP-1 macrophages; and 3) when tested in *in vivo* in fully immunocompetent syngeneic 4T1 tumor mouse model (**Figure 5**), the adoptive transfer of a murine analog of CARIR modified myeloid cells significantly slowed the aggressive 4T1 tumor growth and prolonged survival, but no change of body weight was observed during the experiment as compared to the control groups, suggesting a lack of overt treatment toxicity.

When assessed *in vivo*, the adoptive transfer of CARIR myeloid cells demonstrated significant protective effect, albeit insufficient to fully reverse the disease (**Figure 5**). Several potential factors may account for the observed transient efficacy of CARIR myeloid cells *in vivo*. Firstly, compared to the rapid action by cytotoxic T lymphocytes (CTL) or natural killer (NK) cells, macrophage-mediated phagocytosis or cytotoxicity against tumor cells is inherently slow (26). Given the aggressive nature of the 4T1 cancer model, CARIR-macrophages might struggle to match its pace. Notably, while mouse tumor models progress swiftly, human cancers typically evolve over months or years. Secondly, although 4T1 tumor exhibit positive PD-L1 expression, the levels are relatively moderate (10). Achieving a robust *in vivo* anti-tumor effect with CARIR myeloid cells may necessitate higher levels of PD-L1 expression on target tumor cells. This is suggested by the flow cytometry-based *in vitro* cytotoxicity assay results, where the levels of PD-L1 expression on the target tumor cells correlated with CARIR-mediated elimination by THP-1 macrophages (**Figures 2A–C**). Lastly, there could be temporary or sustained downregulation of PD-L1 expression in 4T1 tumors post-treatment. Such antigen escape mechanisms have been extensively documented in B-cell malignancy subsequent to CAR-T cell therapy (27). However, the natural low affinity at about 7.2 μM between PD-1 (CARIR) and PD-L1 (24), which is several logs lower than typical nM affinity between a CAR and its cognate tumor antigen, make substantial CARIR-mediated trogocytosis of PD-L1 less likely (28). Moreover, the mechanism of action for CARIR macrophages is expected to require recruiting tumor-specific T cells and/or NK cells (29, 30). Once expected tumor antigen cross-presentation has initiated, PD-L1 expression will not be a limiting factor for the anti-tumor effect of CARIR macrophages. Whether the *in vivo* efficacy of CARIR myeloid cells could be further improved warrants further investigation.

In this proof-of-principle study, our focus was primarily on establishing the potential *in vivo* anti-tumor efficacy. Therefore, we did not include any early time points for analyzing the phenotype of the adoptively transferred CARIR myeloid cells within the TME. However, recent reports by others have shed light on the fate of adoptively transferred genetically engineered immature myeloid cells. These cells have been shown to preferentially accumulate at tumor sites, differentiate into proinflammatory M1-like F4/80^+^ macrophages as well as CD11b^-^F4/80^-^CD11c^+^ conventional dendritic cells (cDC), and amount anti-tumor activity through recruiting and activating tumor specific T cells and/or NK cells (29, 30). We hypothesize that a similar mechanism may apply to CARIR macrophages; however, this remains to be determined in future follow up studies. Notably, orthotopically implanted 4T1 mammary carcinoma can spontaneously metastasize to multiple distant sites including liver, lung, and brain (31). In the current study, however, the tumor cells were implanted subcutaneously on the right flank. Gross necropsy was performed in mice that had succumbed to the disease, but no visible metastatic lesions were identified, possibly due to the fast-growing nature of the aggressive subcutaneous 4T1 tumor. Interestingly, a recent pre-clinical study demonstrated that genetically engineered myeloid cells expressing IL-12 could effectively control metastasis through functionally modulate the immunosuppressive pre-metastatic TME (30). Whether CARIR myeloid cells possess such similar effect is to be determined in future studies by employing appropriate tumor metastasis models.

Taken together, we described here an approach employing CARIR modified macrophages as a potential treatment for PD-L1^+^ solid cancer. CARIR expression in macrophages increased phagocytosis and killing of PD-L1^+^ target cells, and adoptive transfer of CARIR transduced myeloid cells slowed progression of aggressive 4T1 tumor and prolonged survival in immunocompetent syngeneic mice. These proof-of-principle results support the utility of CARIR modified autologous myeloid cells to be further developed as a potential therapy for PD-L1^+^ solid cancer.

## Data availability statement

The original data and contributions presented in this study are included in the article. Further inquiries can be directed to the corresponding authors.

## Ethics statement

Ethical approval was not required for the studies on humans in accordance with the local legislation and institutional requirements because only commercially available established cell lines were used. The animal study was approved by Institutional Animal Care and Use Committee (IACUC) of Washington Biotechnology, Inc. The study was conducted in accordance with the local legislation and institutional requirements.

## Author contributions

KC: Formal analysis, Investigation, Methodology, Writing – review & editing. DG: Formal analysis, Investigation, Methodology, Writing – review & editing. BG: Investigation, Methodology, Writing – review & editing. SV: Formal analysis, Investigation, Methodology, Writing – review & editing. MM: Investigation, Methodology, Writing – review & editing. AZ: Conceptualization, Data curation, Formal analysis, Investigation, Methodology, Project administration, Supervision, Validation, Writing – original draft, Writing – review & editing. PA: Conceptualization, Methodology, Project administration, Resources, Supervision, Writing – original draft, Writing – review & editing.

## References

[1] U.S. Department of Health and Human Services Center for Disease Control and Prevention National Cancer Institute. U.S. Cancer Statistics Data Visualizations Tool, based on 2020 submission data (1999-2018). Available online at: https://gis.cdc.gov/Cancer/USCS/#/AtAGlance/.

[2] Morotti M Albukhari A Alsaadi A Artibani M Brenton JD Curbishley SM. Promises and challenges of adoptive T-cell therapies for solid tumours. Br J Cancer. (2021) 124:1759–76. doi: 10.1038/s41416-021-01353-6 PMC8144577 33782566

[3] Klichinsky M Ruella M Shestova O Lu XM Best A Zeeman M. Human chimeric antigen receptor macrophages for cancer immunotherapy. Nat Biotechnol. (2020) 38:947–53. doi: 10.1038/s41587-020-0462-y PMC7883632 32361713

[4] Bauml J Barton D Ronczka A Cushing D Klichinsky M Dees EC. A phase 1, first in human (FIH) study of adenovirally transduced autologous macrophages engineered to contain an anti-HER2 chimeric antigen receptor (CAR) in subjects with HER2 overexpressing solid tumors. Cytotherapy. (2021) 23. doi: 10.1016/s1465324921004205

[5] Vitale I Manic G Coussens LM Kroemer G Galluzzi L. Macrophages and metabolism in the tumor microenvironment. Cell Metab. (2019) 30:36–50. doi: 10.1016/j.cmet.2019.06.001 31269428

[6] DeNardo DG Ruffell B. Macrophages as regulators of tumour immunity and immunotherapy. Nat Rev Immunol. (2019) 19:369–82. doi: 10.1038/s41577-019-0127-6 PMC7339861 30718830

[7] Gordon SR Maute RL Dulken BW Hutter G George BM McCracken MN. PD-1 expression by tumour-associated macrophages inhibits phagocytosis and tumour immunity. Nature. (2017) 545. doi: 10.1038/nature22396 PMC5931375 28514441

[8] Strauss L Mahmoud MAA Weaver JD Tijaro-Ovalle NM Christofides A Wang Q. Targeted deletion of PD-1 in myeloid cells induces antitumor immunity. Sci Immunol. (2020) 5:495–9. doi: 10.1126/sciimmunol.aay1863 PMC7183328 31901074

[9] Wang X Teng F Kong L Yu J. PD-L1 expression in human cancers and its association with clinical outcomes. Onco Targets Ther. (2016) 9. doi: 10.2147/OTT.S105862 PMC4990391 27574444

[10] Sagiv-Barfi I Kohrt HEK Czerwinski DK Ng PP Chang BY Levy R. Therapeutic antitumor immunity by checkpoint blockade is enhanced by ibrutinib, an inhibitor of both BTK and ITK. Proc Natl Acad Sci U S A. (2015) 112:E966–72. doi: 10.1073/pnas.1500712112 PMC4352777 25730880

[11] Morrissey MA Williamson AP Steinbach AM Roberts EW Kern N Headley MB. Chimeric antigen receptors that trigger phagocytosis. Elife. (2018) 7. doi: 10.7554/eLife.36688 PMC6008046 29862966

[12] Paasch D Meyer J Stamopoulou A Lenz D Kuehle J Kloos D. Ex vivo generation of CAR macrophages from hematopoietic stem and progenitor cells for use in cancer therapy. Cells. (2022) 11. doi: 10.3390/cells11060994 PMC8947001 35326445

[13] Blank C Brown I Peterson AC Spiotto M Iwai Y Honjo T. PD-L1/B7H-1 inhibits the effector phase of tumor rejection by T cell receptor (TCR) transgenic CD8 T cells. Cancer Res. (2004) 64:1140–5. doi: 10.1158/0008-5472.can-03-3259 14871849

[14] Peng Q Zhu X Li C Xin P Zheng Y Liu S. APDL1-CART cells exhibit strong PD-L1-specific activity against leukemia cells. Aging. (2021) 13. doi: 10.18632/aging.202578 PMC7993657 33653969

[15] Xie YJ Dougan M Jailkhani N Ingram J Fang T Kummer L. Nanobody-based CAR T cells that target the tumor microenvironment inhibit the growth of solid tumors in immunocompetent mice. Proc Natl Acad Sci U S A. (2019) 116:7624–31. doi: 10.1073/pnas.1817147116 PMC6475367 30936321

[16] Liu M Wang X Li W Yu X Flores-Villanueva P Xu-Monette ZY. Targeting PD-L1 in non-small cell lung cancer using CAR T cells. Oncogenesis. (2020) 9. doi: 10.1038/s41389-020-00257-z PMC7426958 32792499

[17] Qin L Qin L Zhao R Wei X Wu Q Long Y. Chimeric antigen receptor T cells targeting PD-L1 suppress tumor growth. biomark Res. (2020) 8. doi: 10.1186/s40364-020-00198-0 PMC7268496 32514352

[18] Bajor M Graczyk-Jarzynka A Marhelava K Burdzinska A Muchowicz A Goral A. PD-L1 CAR effector cells induce self-amplifying cytotoxic effects against target cells. J Immunother Cancer. (2022) 10. doi: 10.1136/jitc-2021-002500 PMC8796262 35078921

[19] Fabian KP Padget MR Donahue RN Solocinski K Robbins Y Allen CT. PD-L1 targeting high-affinity NK (t-haNK) cells induce direct antitumor effects and target suppressive MDSC populations. J Immunother Cancer. (2020) 8. doi: 10.1136/jitc-2019-000450 PMC7247398 32439799

[20] Qin L Cui Y Yuan T Chen D Zhao R Li S. Co-expression of a PD-L1-specific chimeric switch receptor augments the efficacy and persistence of CAR T cells via the CD70-CD27 axis. Nat Commun. (2022) 13. doi: 10.1038/s41467-022-33793-w PMC9561169 36229619

[21] Liu X Ranganathan R Jiang S Fang C Sun J Kim S. A chimeric switch-receptor targeting PD1 augments the efficacy of second-generation CAR T cells in advanced solid tumors. Cancer Res. (2016) 76:1578–90. doi: 10.1158/0008-5472.CAN-15-2524 PMC4800826 26979791

[22] Kobold S Grassmann S Chaloupka M Lampert C Wenk S Kraus F. Impact of a new fusion receptor on PD-1-mediated immunosuppression in adoptive T cell therapy. J Natl Cancer Inst. (2015) 107. doi: 10.1093/jnci/djv146 PMC4609553 26105028

[23] Herbst RS Soria JC Kowanetz M Fine GD Hamid O Gordon MS. Predictive correlates of response to the anti-PD-L1 antibody MPDL3280A in cancer patients. Nature. (2014) 515:563–7. doi: 10.1038/nature14011 PMC4836193 25428504

[24] Magnez R Thiroux B Taront S Segaoula Z Quesnel B Thuru X. PD-1/PD-L1 binding studies using microscale thermophoresis. Sci Rep. (2017) 7. doi: 10.1038/s41598-017-17963-1 PMC5732298 29247197

[25] Gridelli C Ardizzoni A Barberis M Cappuzzo F Casaluce F Danesi R. Predictive biomarkers of immunotherapy for non-small cell lung cancer: Results from an Experts Panel Meeting of the Italian Association of Thoracic Oncology. Transl Lung Cancer Res. (2017) 6:373–86. doi: 10.21037/tlcr.2017.05.09 PMC5504112 28713682

[26] Pan Y Yu Y Wang X Zhang T. Tumor-associated macrophages in tumor immunity. Front Immunol. (2020) 11:583084. doi: 10.3389/fimmu.2020.583084 33365025 PMC7751482

[27] Mishra A Maiti R Mohan P Gupta P. Antigen loss following CAR-T cell therapy: mechanisms, implications, and potential solutions. Eur J Haematol. (2024) 112:211–22. doi: 10.1111/ejh.14101 37705357

[28] Olson ML Mause ERV Radhakrishnan SV Brody JD Rapoport AP Welm AL. Low-affinity CAR T cells exhibit reduced trogoytosis, preventing rapid antigen loss, and increasing CAR T cell expansion. Leukemia. (2022) 36:1943–6. doi: 10.1038/s41375-022-01585-2 PMC9252916 35490197

[29] Suresh R Barakat DJ Barberi T Zheng L Jaffee E Pienta KJ. NF-kB p50-deficient immature myeloid cell (p50-IMC) adoptive transfer slows the growth of murine prostate and pancreatic ductal carcinoma. J Immunother Cancer. (2020) 8:e000244. doi: 10.1136/jitc-2019-000244 31940589 PMC7057444

[30] Kaczanowska S Beury DW Gopalan V Tycko AK Qin H Clements ME. Genetically engineered myeloid cells rebalance the core immune suppression program in metastasis. Cell. (2021) 184:2033–2052. doi: 10.1016/j.cell.2021.02.048 33765443 PMC8344805

[31] Pulaski BA Ostrand-Rosenberg S. Mouse 4T1 breast tumor model. Curr Protoc Immunol. (2001), 20.2.1-20.2.16. doi: 10.1002/0471142735.im2002s39 18432775

